# Social Marketing Intervention to Engage Older Adults in Balance Workshops for Fall Prevention: A Multicenter Quasi-Experimental Protocol Study

**DOI:** 10.3389/fpubh.2021.614119

**Published:** 2021-07-14

**Authors:** Luc Goethals, Nathalie Barth, David Hupin, Boris Chapoton, Jessica Guyot, Thomas Celarier, Frederic Roche, Karine Gallopel-Morvan, Bienvenu Bongue

**Affiliations:** ^1^Laboratoire SAINBIOSE, U1059 INSERM–Université Jean Monnet, Saint-Etienne, France; ^2^Chaire Santé des Ainés–Université Jean Monnet, Saint-Etienne, France; ^3^Gérontopole AURA, Saint-Etienne, France; ^4^Service de Physiologie, Clinique et de l'Exercice, CHU de Saint-Etienne, Saint-Etienne, France; ^5^Department of Medicine, K2, Solna Karolinska Institutet, Stockholm, Sweden; ^6^Université Lyon, Université Saint-Étienne, HESPER EA 7425, Saint-Etienne, France; ^7^Service de Gérontologie Clinique, CHU de Saint-Etienne, Saint-Etienne, France; ^8^Ecole des Hautes Etudes en Santé Publique/EA 7348 MOS, Rennes, France; ^9^Centre Technique d'Appui et de Formation, Saint-Etienne, France

**Keywords:** older adult, social marketing, prevention, fall, intervention

## Abstract

**Background:** Falls affects one of three people after 65 years old, and it can lead to serious consequences. Scientific evidence point out that physical exercise is the most efficient way to prevent falls among older adults.

**Objective:** The main objective of this study is to determine if a social marketing program can increase the attendance rate of people aged 60 and over at group balance workshops.

**Methods:** This quasi-experimental multicenter study is being conducted in three French Regions (Loire, Haute-Loire and Rhône) over a period of 18 months. The Social Marketing Campaign will be done in three ways. Firstly, a Communication Campaign will take place in the two Test Areas but not in the Control Area. Secondly, flyers have been designed to be distributed by local partners. Finally, conferences for older people will be organized in the areas of intervention in order to reach the target audience for the program. The study will include people aged 60 and older who want to participate in the Balance Program.

**Results:** The Crédit Agricole Loire/Haute-Loire Foundation funded the study and the Jean Monnet University of Saint-Etienne reviewed it. The Ethics Committee of the University Teaching Hospital of Saint-Etienne approved and peer-reviewed it on September 6, 2019, under Reference Number IRBN622019/CHUSTE.

**Conclusion:** The results of this first study will demonstrate whether or not social marketing for promoting group balance workshops in the elderly will increase their attendanceship in adapted physical activity sessions, especially those that prevent falls.

**Clinical Trial Registration:** https://clinicaltrials.gov/ct2/show/NCT04136938, identifier NCT04136938.

## Background

The global phenomenon of aging present several public health challenges.; namely the development of long term care, the creation of adapted environments for elderly (1). Projections show between 2015 and 2050, that the proportion of over-60-year-olds in the world population will increase from 12 to 22%, representing about 2 billion people (1). Life expectancy is increasing, and should be accompanied with the improvement of the quality of life through the preservation of functional activity, i.e., physical activity. It is important though to allow older adults to maintain their autonomy (2, 3).

Falls remain a significant source of morbidity in people aged 65 in one out of three in this age group (4). The association between PA and the risk of falling in elderly was recently evaluated by systematic review and meta-analysis. These analyses came out with the conclusion that the risk of being a recurrent faller (two or more self-reported falls over the follow-up period of 12–36 months) was 39% higher in older adults with the lowest levels of PA (5).

Therefore, physical activity may be the most effective method in preventing falls among older people (4, 6, 7). Meanwhile the interventions aimed at maintaining balance are the most effective to avoid loss of autonomy (4, 8). Obviously the programs based on the maintenance of the balance are the least expensive There is evidence that these balance-based programs can be cost-effective (9); They produce great health results and particularly improve the quality of the rest of participants life (4, 9, 10). A 2019 meta-analysis (4) confirmed and supported that physical activity is indeed one of the most effective techniques for reducing fall rates and risk of falling. In this meta-analysis, 108 randomized controlled trials were included, with 23,407 participants in 25 countries. Programs offering multiple types of exercise (balance, functional and resistance) reduced the rate of falls by 34% and the number of people who fell by 22%, according to the authors. When interventions offered different forms of physical activity, including balance exercises, the rate of falls, compared with the no-intervention groups, was reduced by 24%, and the number of people who had one or more falls by 13%.

However, although there now is unanimity over its practical usefulness, there are many barriers in getting the elderly to participate in any PA (11, 12). Indeed, some believe they are no longer able to do so because of a loss of physical capacity, while others find that sports is only for the young or healthy. All this is because of the little awareness of the tailored activities that exist (11–13). That maybe the reason why, sum all, only a small number of elder participate in PA for fall prevention (14, 15).

The Balance Workshops, such as those developed by the French Federation of Physical Education and Voluntary Gymnastics (FFPEVG), aim at preserving the balancing function and the autonomy of older people. They also aim at giving them confidence in their movements, reducing the psychological impact of falling, boosting their self-esteem and increasing their mobility. This Balance Program will last for 12 weeks, including ten sessions of group PA and two individual test sessions.

Through these sessions, the sports trainer, specialized in the approach, will engage them in various motor movements that involve the different components of balancing. Depending on the capacities of the various participants and the results obtained during the tests, the sports trainer will design educational approaches that address the different components of balancing. There is the fieldwork that consists of collecting information and managing the environment, finding a place, applying methods, which lead to loss and recovery of balance, carrying out activities for muscular, and kinesthetic strengthening, and exercises tied to walking (rhythm, frequency, overcoming obstacles). There is also a global approach to handling the matter of balance in the form of advice – this by including themes on aging well, such as the impact of drugs, food, and home improvement (16).

Introduced in 1971 by Kotler and Zaltman (17), social marketing is used extensively by health actors and researchers in several areas such as smoking prevention, promotion of healthy eating and physical activity (18–22). Alain Andreasen in 1995, defined social marketing as “*the adaptation of commercial marketing technologies to programs designed to influence the voluntary behavior of target audiences to improve their personal welfare and that of the society*” (23). Therefore, social marketing, intends to appropriate the principles and approaches of commercial marketing (market research, targeting and segmentation of the target audience, the 4Ps of marketing: product, place, price and promotion) particularly used by private companies to promote prevention programs (24). The Social Marketing method is based on seven benchmark criteria defined by researchers in the field (25–28), specifically including:

- Behavioral objective: Defining a behavioral change objective for the intended target audience is fundamental, especially for evaluating the effectiveness of an implemented program (29, 30) (i.e., to increase PA);
- Formative research: understanding the target audience is based on conducting market research. Market research provides an in-depth understanding of the needs, desires, values and lifestyles of the target audience (31). The purpose of market research is to inform the planning, development and implementation of social marketing programs (32, 33);
- Segmentation: Segmenting means creating homogeneous groups of individuals with identifiable similarities. These similarities can be, for example, place of residence, age, lifestyle or health status (34, 35);
- Exchange: To induce individuals to change their behavior, it seems important to provide them with something in exchange. R. Bagozzi assumes that the exchange between several social actors (e.g., the target audience and the promoters of a prevention programme) involves a transfer of tangible or intangible objects (36);
- Marketing Mix: Social marketers have therefore taken the “4Ps” concept from commercial marketing and adapted it (88, 89, 111): Product: the behavioral offer made to the target audience. The product involves intangible elements such as the adoption of an idea or behavior; Price: offering financial support mechanisms to address financial or psychological barriers to adopting the proposed behavior; Place: facilitating access to services or products that enable a change toward the proposed behavior; Promotion: promoting the behavior through the use of communication tools;
- Competition: In social marketing, as in commercial marketing, practitioners need to be aware that there are sources of competition to the adoption of the proposed behavior (23, 30). The main source of competition is the tendency of the target audience to maintain their current behavior, especially when that behavior involves an addiction (tobacco, alcohol, drugs, etc.) (37).
- Evaluation: An assessment of the program is carried out.

Social marketing principles for preventional intervention have been successfully used in various field, including smoking prevention, healthy eating promotion, alcoholism reduction, and physical activity promotion (13–15). Furthermore, these social marketing initiatives have been effective in various age groups, from adolescents (38), adults (39) to older adulsts (40).

Although this method has proven its worth in these other contexts, its use in getting older adults to take part in regular PA remains limited (41).

The main objective of this study is to determine whether associating a Social Marketing program would increase the attendance rate of people aged 60 and over in Group Balance Workshops.

The secondary objectives of this study are: (1) to increase the participation rate in the Balance Workshop sessions, (2) to increase the level of the quality of life of the participants, and (3) to increase the level of PA among the people participating in the study.

## Materials and Methods

### Design and Setting (Study Status)

This quasi-experimental multicenter study is being conducted in three French Regions (Loire, Haute-Loire and Rhône). The quasi-experimental model was decided upon in view of testing causal hypotheses. It is envisaged that the program will be evaluated using a set of indicators that have been predefined.

The distribution of participants between the two groups (Test and Control) was based on the choice of the study designers. A Comparison group that is as close as possible to the Test group in terms of initial characteristics will allow us to identify the outcomes in the case where the Social Marketing campaign is not implemented. This method will bring proof as to whether the program will be the cause of the differences in outcomes between the Test and Comparison groups.

**Figure 1:**
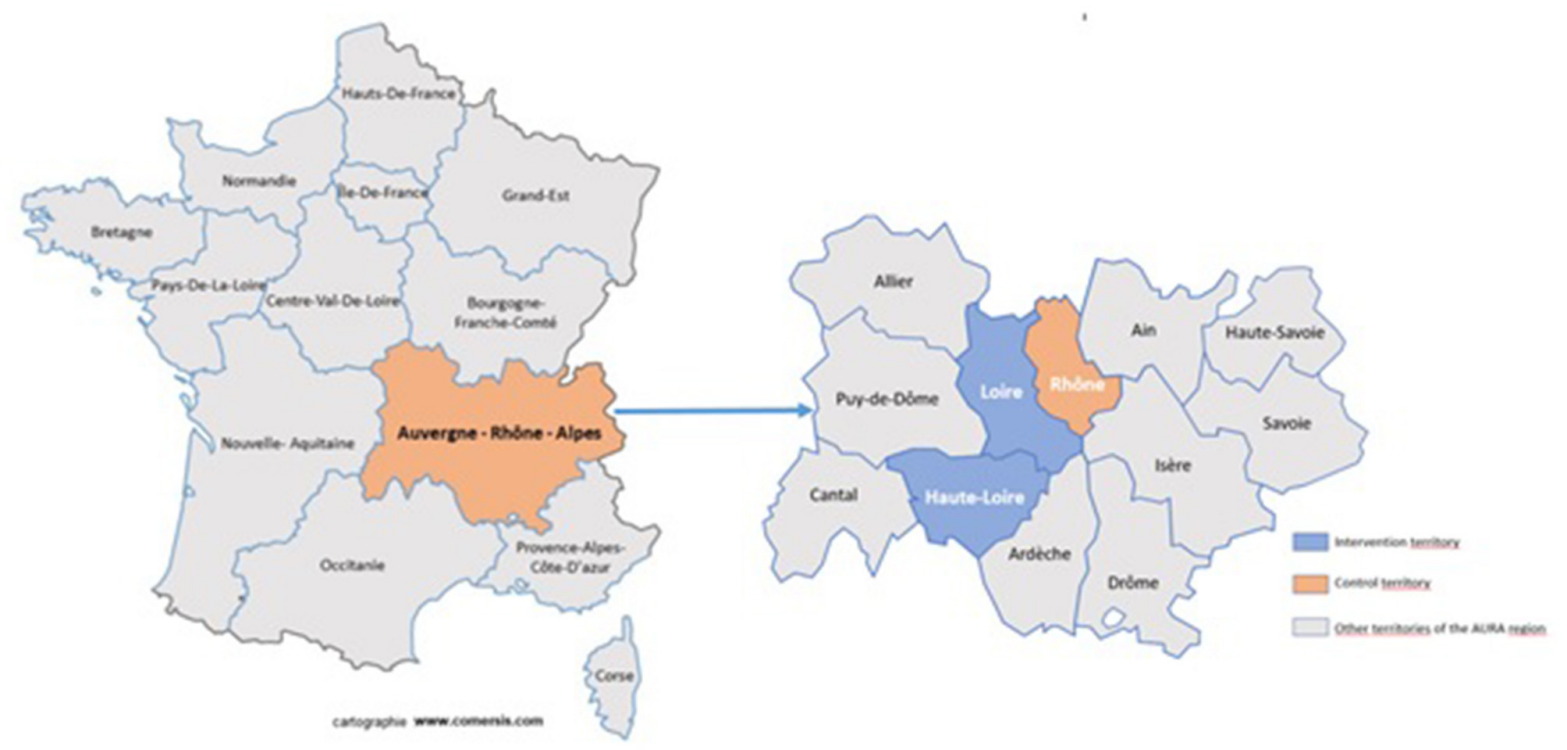
Test and control areas of the study.

The Loire and Haute-Loire Regions were selected for the campaign to promote the Balance Workshops against falls in the older population. This was because of the presence of our partners for the implementation of the study. The Rhône Region was selected, not as a Test, but as the Control Area (Figure 1).

The Social Marketing Campaign consists of three ways; firstly, by a Communication Campaign in the two Test Areas using advertisements developed and posted in the local print media. Secondly, flyers have been designed to be distributed by our local partners. Finally, conferences for older people will be organized in the test areas to reach the target audience for this program.

**Figure 2:**
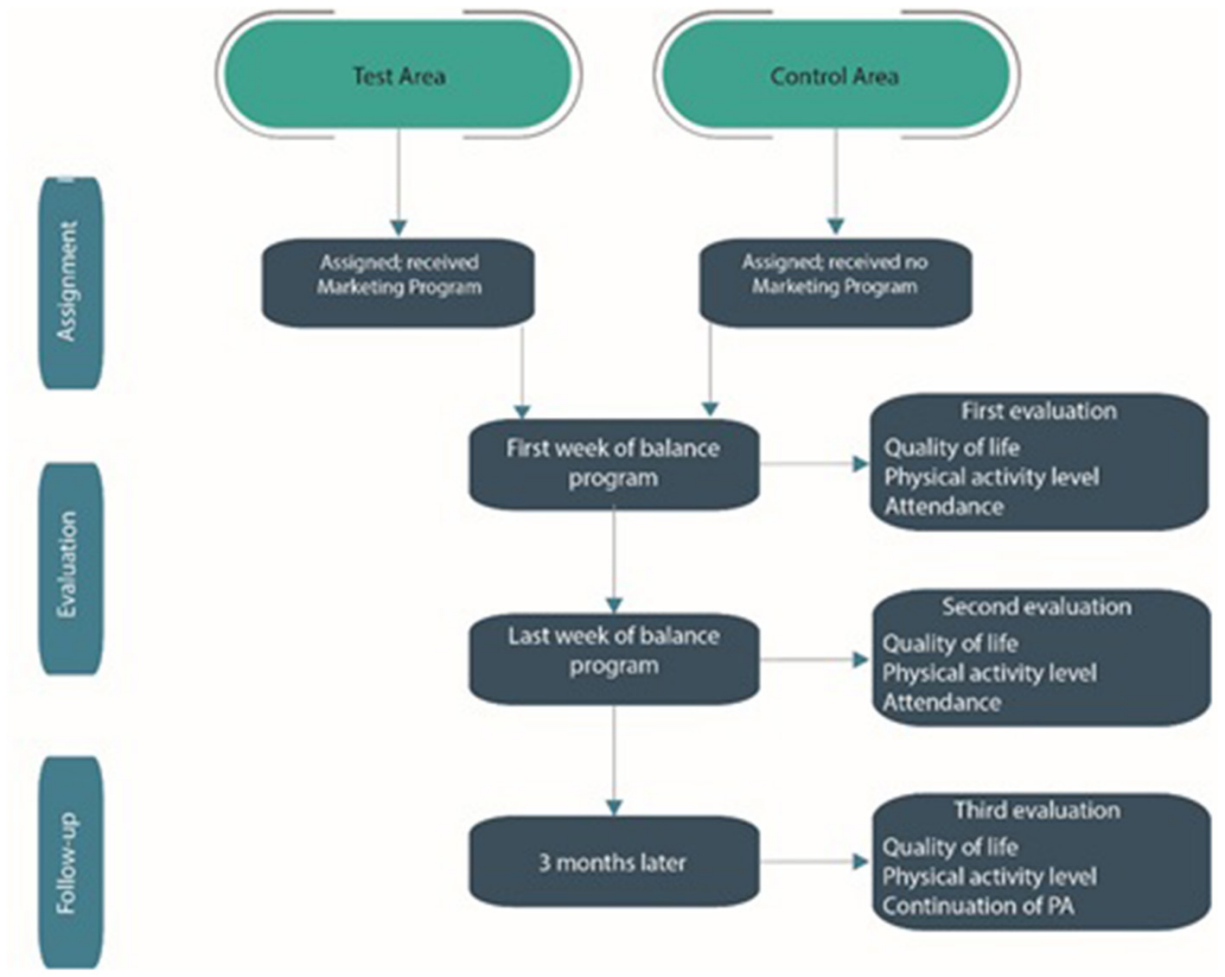
Flow-chart of the study.

Finally, to encourage people to participate in this PA program, a gift card worth 20 € will be distributed to participants at registration. Then, a second gift card worth € 20 will be distributed to those who carry out at least 10 sessions out of the 12 offered. This study will last for 18 months. The study is underway and started in March 2020. The study flow-chart (Figure 2) summarize the study.

### Recruitment in the Test Area

#### Selection

Individuals in the study areas who have seen the advertisement for the Balance Workshops and who fulfill the inclusion criteria will be eligible for the study. They will contact our partners (“Atouts Prévention” and “Association Régionale Santé Prévention sur les Territoires Auvergne” through the contact details provided alongside the advertisements) to register for the workshops organized by the French Federation of Physical Education and Voluntary Gymnastics (FFPEVG) at the local level.

#### Registration

During the first session, each study participant will be given an Information Sheet as well as a 20 € gift card. A computerized data collection tool designed for the study and allowing for the constitution of the database will be available during the collection phase from the first session of the workshops and then 6 months after inclusion.

#### Follow-Up

Those who consent to participate in the study will go through all the sessions for the 12 weeks. Gift cards will be distributed to those who complete 10 of the 12 sessions. During the 1st and 12th sessions, the FFPEVG organizers will collect the previously mentioned data from the study participants. Finally, 3 months after the end of the Balance Workshop sessions, participants will be contacted by the Principal Investigator by telephone to collect the same data.

### Recruitment in the Control Area

#### Selection

In the same way as when registering people for the Balance Workshops, the FFPEVG of the Rhône will contact older people who meet the inclusion criteria.

#### Registration

During the first session, each study participant will be given an Information Sheet. A computerized data collection tool designed for the study and allowing for the constitution of the database will be available during the collection phase from the first session of the workshops and then 6 months after inclusion.

#### Follow-Up

Those who consent to participate in the study will go through all the sessions for the 12 weeks. During the 1st and 12th sessions, the FFPEVG organizers will collect the previously mentioned data from the study participants. Finally, 3 months after the end of the Balance Workshop sessions, the Principal Investigator will contact participants by telephone to collect the same data.

#### End of Study

The end of the study for the participants will be marked by the completion of the telephone interview 3 months after the end of a cycle of Balanced Workshops.

##### Duration of Participation in the Study

An average of 6 months per participant.

#### Inclusion Criteria

- Individuals aged 60 and over
- Individuals living in the Loire, Haute-Loire or Rhône Regions of France
- Individuals who do not have medical contraindications concerning participation in moderate PA
- Individuals who have been fully informed about the study, including their rights over the data to be collected

#### Non-inclusion Criteria

- Persons unfit to participate in Balance Workshop sessions
- Persons already enrolled in a similar PA

#### Partial or Permanent Exclusion Criteria

All participants will be free to cancel the full recording of their data or stop some particular recording in progress. This will be considered as change of opinion or refusal of data transfer. In such a case, the data will not be taken into account and the participant will be classified among the premature stopping of the study by withdrawal.

#### Evaluation Criteria

The primary endpoint is the attendance rate of the participants in the Balance Workshop sessions for fall prevention. The secondary evaluation criteria are the number of calls received for a registration request, the evolution of the level of the quality of life using the SF 36 questionnaire (42, 43) and the evolution of the level of PA of the participants using the short version of the IPAQ questionnaire for older adults (44).

#### Recorded Variables and Source Data

Data will be collected directly from the questionnaires as the study progresses.

The information collected on the participants will include:

- Sociodemographic characteristics: Age, subjective age, sex, level of education, family situation, living conditions
- Attendance and participation rates in the workshops
- How they got to know about the Balance Workshops.
- Individual Precariousness Index - Evaluation of the Deprivation and Inequalities of Health in Healthcare Centers (EPICES) Score: This assessment is made up of 11 questions that summarize the precarious situation of a person. A coefficient is assigned to each response, the sum of which gives the EPICES score which is continuous, and varies from zero (lack of precariousness) to 100 (maximum precariousness). The evaluation of this score has been relevant in detecting and quantifying precariousness. A value of 30 is considered as the Precariousness Threshold according to EPICES (45).
- Level of quality of life: Questionnaire SF 36 is the generic questionnaire most often used in Medicine for the evaluation of the quality of life in general. It analyzes the physical, emotional and mental dimensions. The full version includes 36 questions (42, 43). A score for each dimension of the SF-36 is calculated, ranging from 0 to 100. Poor health, loss of function, and presence of pain were scored as low. Good health, absence of functional deficit and pain were scored as high.
- Level of PA: IPAQ Questionnaire - E is a short version of the International Physical Activity Questionnaire for older adults. It was validated for the assessment of the level of PA in older adults and has seven questions that classify them at various levels of PA (44). Official IPAQ guidelines (46) are as follows: data are added “*within each item (i.e., vigorous intensity, moderate intensity, and walking) to estimate the total amount of time spent engaged in PA per week. Total weekly PA (MET-min week*−*1) was evaluated by adding the products of reported time for each item by a MET value, specific to each category of PA*” (46). “*MET values were assigned as projected for use with elderly adults: vigorous PA* = *5.3 METs, moderate PA* = *3.0 METs, and walking* = *2.5 METs*” (47).

## Statistical Analyses

### Number of Participants Required

In the Balance Workshops carried out by the “Caisses d'Assurance Retraite et de la Santé au Travail” (CARSAT) for the Loire and Rhône Regions in 2018, about 43% of those who registered went through the 12 sessions. Attendance was at 44% in the Loire Region and 42% in the Rhône Region. In our study, we hope to raise the attendance by about 20%, to get to 63% per group who go through the 12 sessions.

We compared two binomial proportions observed with:

$$\begin{array}{l} \text{π}1=0\text{.}43\text{ π}2=\;0\text{.}63\\ \text{α}=5\%\;Power1-\text{β}=\;0\text{.}9 \end{array}$$

The total number of participants required is 210 (with 105 for the Test Area and 105 for the Control Area).

### Description of Statistical Methods

The analyses will be carried out on the entire study population and also by groups, depending on whether they received the specific advertisement materials or not.

#### Univariate Analyses

Descriptive analyses of the variables collected on the study population; comparison of the participants according to their groups using parametric or non-parametric tests according to the distribution of the variables (Chi-Square Test for quantitative variables, Student Test for qualitative variables, and the significance of the tests at 5%).

#### Multivariate Analyses

Analysis of Variance (ANOVA), Generalized Linear Model (GLM), Mixed Model, analyses of multiple components to identify groups of individuals (the study of their profiles).

### Evaluation of the Social Marketing Benchmark Criteria

The Table 1 present the definition and the evaluation of each social marketing benchmark criteria.

**Table 1:** Definition and evaluation of each social marketing benchmark criteria.

**Social marketing steps**	**Définition**	**Evaluation**
Behavioral objective	Defining a behavioral change objective for the intended target audience is fundamental, especially for evaluating the effectiveness of an implemented program	Statistical analysis about an increase or not of PA participation and level
Formative research	Understanding the target audience is based on conducting market research. Market research provides an in-depth understanding of the needs, desires, values and lifestyles of the target audience. The purpose of market research is to inform the planning, development and implementation of social marketing programs	We did this step 1 year before the beginning of the study and allow us to build the research protocol.
Segmentation	Segmenting means creating homogeneous groups of individuals with identifiable similarities. These similarities can be, for example, place of residence, age, lifestyle or health status	Subgroup analysis, particularly by age group, will allow us to know if this segmentation criterion was effective. The qualitative survey of participants in the study areas (control and intervention) will allow us to know if there was any pollution of the study due to the geographical proximity of our territories.
Exchange	To induce individuals to change their behavior, it seems important to provide them with something in exchange. R. Bagozzi assumes that the exchange between several social actors (e.g., the target audience and the promoters of a prevention programme) involves a transfer of tangible or intangible objects (36);	An intervention territory will offer the workshops for 0€ and another territory for 20€. The evaluation of the level of participation by adjusting this parameter will allow us to evaluate the relevance of the free workshops.
Marketing mix	Social marketers have therefore taken the “4Ps” concept from commercial marketing and adapted it (88, 89, 111): Product: the behavioral offer made to the target audience. The product involves intangible elements such as the adoption of an idea or behavior; Price: offering financial support mechanisms to address financial or psychological barriers to adopting the proposed behavior; Place: facilitating access to services or products that enable a change toward the proposed behavior; Promotion: promoting the behavior through the use of communication tools;	The qualitative survey among the participants of the study will allow us to know the relevance of the actions proposed for the four components of the marketing mix.
Competition	In social marketing, as in commercial marketing, practitioners need to be aware that there are sources of competition to the adoption of the proposed behavior. The main source of competition is the tendency of the target audience to maintain their current behavior, especially when that behavior involves an addiction (tobacco, alcohol, drugs, etc.).	The qualitative survey of participants will allow us to assess the influence of other physical activity providers on the decision to participate or not in the proposed program.
Evaluation	An assessment of the program is carried out	We will conduct a statistical evaluation as well as a qualitative survey

### Monitoring

No extra examinations or treatment besides traditional practices will be done; except the digitization of all findings for analysis. During analysis, data will be kept confidential and anonymous. No intervention will be done outside of normal practice. For participants, we do not forsee any unusual risk or specific constraint.

### Ethical Considerations

The “Crédit Agricole Loire/Haute-Loire” Foundation funded the study and the Jean Monnet University of Saint-Etienne reviewed it. The Ethics Committee of the University Teaching Hospital of Saint-Etienne approved and peer-reviewed the study on September 6th, 2019, under Reference Number: IRBN622019/CHUSTE.

Data collection will start in September 2020 and the planned end date for this activity is July 2021. Data analysis will take place in the summer of 2021 and the first results are expected to be published in late 2021.

All participants will receive information brochures, and give their oral informed consent (because of more difficulties in seeing and writing in older adults).

Approval for important protocol modifications will be sought from the Ethics Committee as the need may arise. There are no anticipated risks associated with participation in the study. Patients will be free to withdraw at any time. Data will be kept confidential and anonymized for the analyses.

### Access to Data

Accessibility to cleaned data to all Principal Investigators will be guaranteed. Project data sets will be kept on the LimeSurvey database created for the study, and the data sets will be accessed through a password. Project Principal Investigators will have unlimited access to their own site's data sets, but will need a permission to have access to other sites'data. In order to re-inforce the confidentiality, data shared with project team members will be blinded.

### Publication Policy

The authors will review all publications following the guidelines given below.

#### Data Analysis and Release of Results

The scientific integrity of the project requires that sites'data be pooled before analysis and reported as such. Presentations and publications from the project will be expected to protect the integrity of the major objective(s) of the study. Presented data should not break the blind prior to the release of the endpoint data.

#### Review Process

Each paper or abstract, as described below, must be submitted for review of its appropriateness and scientific merit prior to submission.

#### Primary Outcome Papers

The primary outcome papers are publications that present outcome data, as decided by authors.

#### Other Study Papers, Abstracts and Presentations

Within this category fall all studies except those designated as “Primary Outcome.” Authors should all approved a paper or abstract before submission.

In case of calls to contribution (papers, workshops, symposia, volumes, etc) in respect to related studies, authors may select other team members to bring in their contribution. If time permits, this call may be shared with all team members.

### Reproducible Research

Data sharing statement: No later than 3 years after the collection of the year-1 data, they will be deposited, coded and anonymous in an appropriate data archive for sharing purposes.

## Discussion

Our study will assess whether a Social Marketing approach is more effective in encouraging older adults to participate in Balance Workshops to prevent falls and to be diligent throughout the program. In 2017 in France, more than 76% of everyday accidents among people aged 65 and over were falls (48). Group physical activity offers exist in the country and are very often free. However, few older people participate in such programs. Strategies to increase PA levels, including improving balance, are therefore of particular importance.

Different methods are available for professionals to use to increase PA among older adults. social marketing is used extensively by health actors and researchers in several areas such as smoking prevention, promotion of healthy eating and physical activity (18–22). Prevention interventions, based on social marketing principles, have been successfully carried out in different (13–15) and age groups (38–40).

Our study used the classic and widely-cited Social Marketing benchmark criteria as defined by Andreasen (30) and Gallopel-Morvan et al. (28), including the evaluation as a major step in the process (a seventh criterion) (28). However, it is difficult to evaluate the efficiency of Social Marketing in promoting PA among seniors. Few strategies used so far did not apply all the seven benchmark criteria (49). Fujihira et al. (41) and Goethals et al. (49) have showed that as a program frequently uses the social marketing benchmark criteria defined by Andreasen, it becomes more and more effective at changing the behaviors of its target population. Other reviews (18, 19, 50) have used the same classification criteria as Andreasen and reported similar findings. Social Marketing may therefore be potentially efficient in promoting PA among seniors (49). Given its increased use in such initiatives (51), we aimed at confirming its effectiveness among older adults.

This study brings together a provider (the FFPEVG) and promoters (“Atout Prévention Rhône-Alpes” and ARSEPT Auvergne) which, in this case, are medico-social groups. Currently, the locations where the Balance Workshops take place belong neither to the provider nor to the promoters. To facilitate the setting up of these structures in our areas of intervention, we established collaborations with the City or Community Social Action Centers, which have rooms and gymnasia for such purposes. This joint collaboration between these various groups in these regions will provide a global solution to encourage older adults to move toward PA centered on balance.

Accessibility to the workshops, whether physical and/or financial, is an essential condition for the participation of older adults in these activities (52). This study will lead to the establishment of new workshops in all these areas of intervention, thus making the product more accessible to the target audience. Also, even if this Balance Workshop program is free in one region and costs very little in another, we wanted to offer incentives to be able to deepen our knowledge of their capacity or not to increase participation in our program.

The adaptation of the program to the needs and expectations of the older adults is also a condition that favors their participation (52). That is why we interviewed some of them to identify these issues and improve on our program. From the survey, we developed an intervention strategy that included appropriate communication with them (modification of communication tools and use of new media validated by them) and training of professionals with particular emphasis on the fears and expectations of their trainees.

We equally chose geographic segmentation. The Loire and Haute-Loire (Test) Regions will receive the marketing campaign, but not the Control Area (Rhône Region). These are neighboring Regions, so this introduces a bias in our study. Therefore, those who will run the program particularly in the Control Area, may have to make more efforts to increase participation in their workshops. Besides, local newspapers that are distinct for each area are being used to promote the workshops. However, these older adults move around; and so, some who live in the Control Area may become aware of the campaign and may want to participate in the workshops. There is therefore a risk of contamination of the study.

Finally, though this study focuses on the evaluation of the whole intervention, stepwise evaluations will have to be carried out. For a start, an evaluation of the effectiveness of the campaign will be undertaken through the collection of data on the intention to participate in the study and to attend the workshops. Alongside, an economic evaluation will have to be done to know the most cost-effective and appropriate method of communication through which to encourage older adults to commit themselves to the program. A statistical evaluation will also have to be conducted to first of all highlight whether or not the campaign is more effective in the Test Area than in the Control Area. Then, in a second step, it will assess the evolution of the quality of life with PA levels as a function of exposure to the campaign. Lastly, a qualitative evaluation of the campaign will need to be carried out on the older adults and also on the actors in the field. This study will therefore evaluate the effectiveness of this Social Marketing approach and, if positive, will maximize the probability of the measures applied to be more sustainably adopted in the community.

However, this quasi-experimental model has some limitations. Just like for any technique applied only at the end of a program, the method can be significantly hampered by the lack of baseline data. Since such methods are based on certain causal assumptions, conclusions on causality are less pertinent/persuasive than those resulting from a randomized controlled trial.

## Conclusion

The results of this first study will demonstrate whether or not social marketing for promoting group balance workshops in the elderly will increase their attendanceship in adapted physical activity sessions, especially those that prevent falls.

## Data Availability Statement

The original contributions presented in the study are included in the article/supplementary material, further inquiries can be directed to the corresponding author.

## Ethics Statement

The studies involving human participants were reviewed and approved by Ethics Committee of the University Teaching Hospital of Saint-Etienne. Written informed consent for participation was not required for this study in accordance with the national legislation and the institutional requirements.

## Author Contributions

LG and BB mounted the protocol and wrote the manuscript. BC and KG-M participated in mounting the protocol and corrected the manuscript. All authors have read and approved the final manuscript.

## Conflict of Interest

The authors declare that the research was conducted in the absence of any commercial or financial relationships that could be construed as a potential conflict of interest.

## Figures and Tables

**Table:** 

**Data category**	**Information**
Primary registry and trial identifying number	ClinicalTrials.gov NCT04136938
Date of registration in primary registry	21 October, 2019
Secondary identifying numbers	IRBN622019/CHUSTE
Source(s) of monetary or material support	Université Of Saint-Etienne
Primary sponsor	Fondation Crédit Agricole Loire, Haute-Loire
Secondary sponsor(s)	French Federation of Physical Education and Voluntary Gymnastics
Contact for public queries	*Goethals Luc, 0699222119, luc.goethals@univ-st-etienne.fr*
Contact for scientific queries	Université Jean Monnet, Saint-Etienne, France
Public title	Social marketing intervention to engage older adults in balance workshops for fall prevention
Scientific title	Social marketing intervention to engage older adults in balance workshops for fall prevention: A multicenter quasi-experimental protocol study
Countries of recruitment	France
Health condition(s) or problem(s) studied	Engaging older adults in Physical Activity
Intervention(s)	Intervention area: *social marketing campaign* Control area: without social marketing campaign
Key inclusion and exclusion criteria	- People aged 60 and over - People living in the one of the territories of the study - People without medical contraindications to participate in moderate physical activity - People who has received information about the study and its rights to its data - Exclusion criteria: - People unable to attend workshops - People already registered for adapted physical activity
Study type	Observational Study Model: case control
Date of first enrolment	November 2020
Target sample size	250
Recruitment status	Recruiting
Primary outcome(s)	The increase of the attendance rate of participants
Key secondary outcomes	The increase of the participation rate in fall prevention workshop sessions The increase of the life quality of participants The increase of the physical activity level of participants
